# Nanocarrier-mediated drug delivery systems for spinal cord injury treatment

**DOI:** 10.3389/fbioe.2025.1660264

**Published:** 2025-09-05

**Authors:** Yajun Cheng, Rui Wang, Xiaoyi Zhou, Hao Jiang, Ming Li, Xianzhao Wei

**Affiliations:** ^1^ Department of Orthopaedics, First Affiliated Hospital, Naval Medical University (Second Military Medical University), Shanghai, China; ^2^ Department of Biochemistry and Molecular Biology, College of Basic Medical Sciences, Naval Medical University, Shanghai, China; ^3^ Spine Tumor Center, Changzheng Hospital, Naval Medical University, Shanghai, China

**Keywords:** spinal cord injury, nanocarriers, drug delivery, targeted therapy, nanoparticles and nanomaterials

## Abstract

Spinal cord injury is a severe neurological condition that frequently. Results in lasting motor and sensory dysfunction. Traditional drug therapies have shown limited efficacy in addressing the complexities of spinal cord injury. This limitation highlighting the urgent need for innovative treatment strategies. In recent years, nanocarrier-mediated systems have garnered significant attention due to their superior drug delivery capabilities and targeting precision. This review summarizes the latest advancements in the application of nanocarriers for the treatment of spinal cord injuries, discussing various types of nanocarriers, drug loading and capacity and release profiles, as well as targeted delivery strategies. The insights aim to establish a theoretical foundation for future research and clinical applications in this critical area of medicine.

## Introduction

**FIGURE 1:**
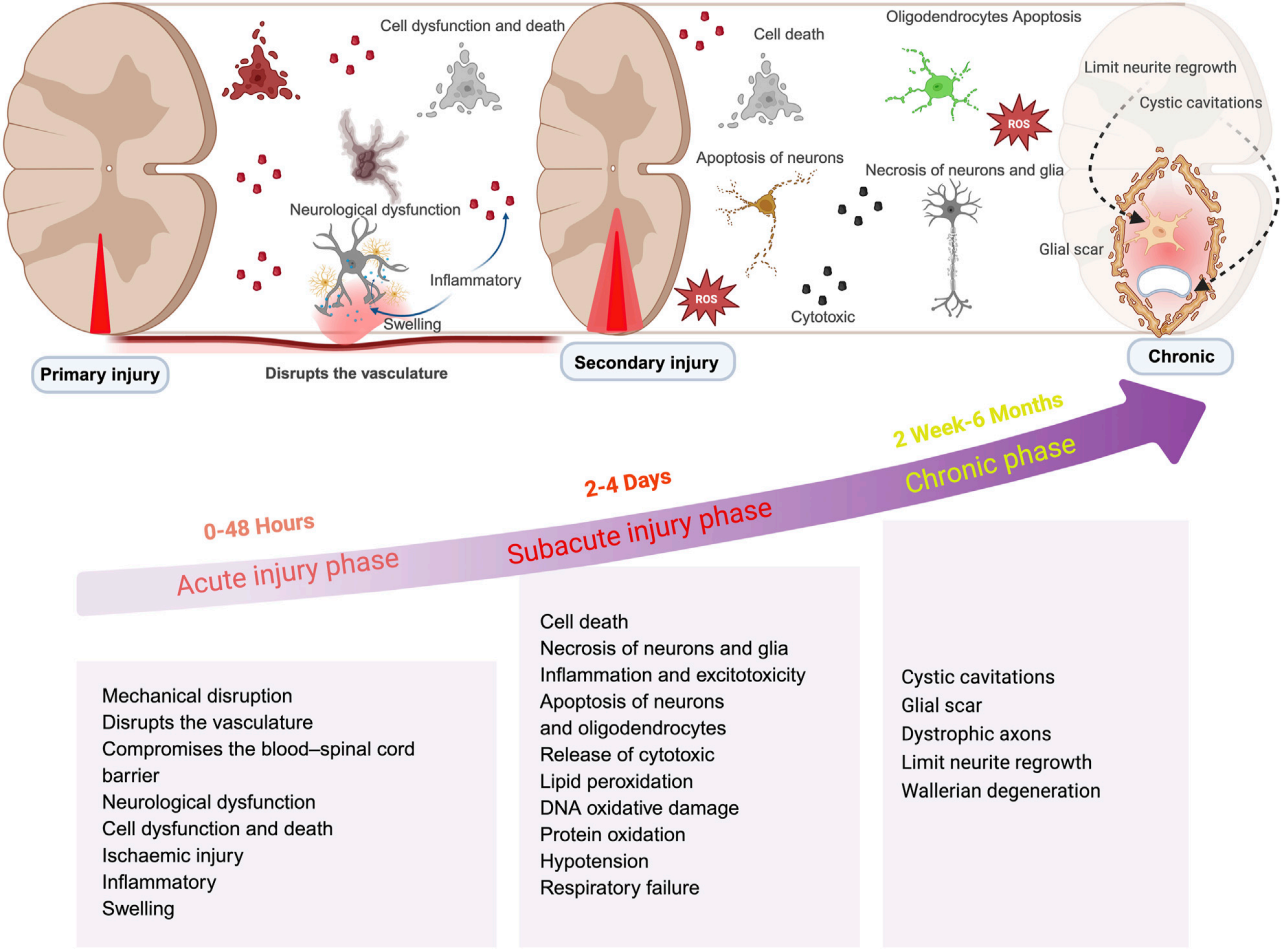
Schematic illustrations of changes in spinal cord injury at different phase. This illustration is organized into three distinct stages: acute, subacute, and chronic. The acute phase, occurring within 0–48 h, is characterized by cascades of secondary injury; the subacute phase, spanning from 2 to 4 days, is defined by prolonged inflammation; while the chronic phase, extending beyond 2 weeks, is identified by the maturation of glial scars and additional pathological alterations. (created with https://biorender.com).

Spinal Cord Injury (SCI) is a traumatic disruption of spinal cord integrity, resulting in transient or permanent functional alterations of motor, sensory, and autonomic nervous systems, with profound consequences for patient quality of life (Eckert and Martin, 2017; McDonald and Sadowsky, 2002). As the most prevalent cause of disability among spinal disorders, SCI pathophysiology comprises two distinct phases:primary injury from mechanical trauma and subsequent secondary injury driven by molecular cascades (Jendelova, 2018; Chay and Kirshblum, 2020). In most injuries the spinal cord is compressed and the extent of the damage depends primarily on the force of the compression directly causing damage to the spinal cord tissue and nerve cells necrosis (Zheng and Tuszynski, 2023; Dams-O'Connor et al., 2023). Secondary injury refers to additional damage caused by local ischemia, edema, and inflammation. These processes affect the normal tissues surrounding the lesion. This impairs the normal delivery of nutrients and oxygen to cells, leading to the release of toxic chemicals for excitotoxicity, disrupting the integrity of adjacent cells, and further spreading and exacerbating the spinal cord injury (Ambrozaitis et al., 2006; Sterner and Sterner, 2022; Gao W. et al., 2022) (Figure 1). SCI can cause fatal or long-term disabilities, including paralysis, sensory loss, organ dysfunction, mental disorders, and behavioral complications such as drug abuse and self-injury, which greatly affect the patient’s quality of life. (Anjum et al., 2020). SCI also has varying degrees of impact on a patients’ occupation, family, and economy (Radhakrishna et al., 2014; Malekzadeh et al., 2022). Therefore, effective treatment of SCI is crucial. Even with the continuous efforts of scientists, although many neuroprotective and neuroregenerative therapies have entered preclinical trials, there are currently no approved drugs for the treatment of spinal cord injury. While nano-drug delivery systems hold great potential in spinal cord injury treatment, further research is needed to optimize design, improve delivery efficiency, and ensure safety. Future research and clinical trials should validate the application prospects of nano-drug delivery systems in spinal cord injury treatment.

**FIGURE 2:**
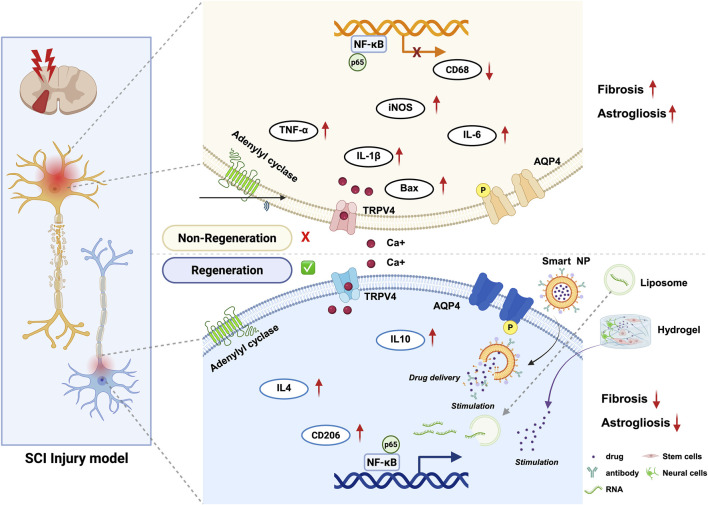
Schematic overview of Nanocarrier-mediated delivery strategies to treat spinal cord injury. Nanocarrier -mediated delivery strategies include the delivery of bioactive substances regulating and cell therapy, applied from different perspectives to repair spinal cord injury and associated molecular changes. (created with https://biorender.com).

The field of spinal cord injury treatment has seen the application of nanomedicine technology in recent years, which brings new hope for spinal cord injury treatment (Wang et al., 2024; Liang et al., 2024; Prakash, 2023). Nano-drug delivery systems utilizes nanoparticles to deliver therapeutic drugs to the spinal cord injury site in a targeted manner. This systems have advantages of: controlled release, enhanced drug stability, breaking through the blood-brain barrier, and combination therapy (Gao et al., 2016; Meyer et al., 2015; Feliu and Parak, 2024). These nanocarriers can enhance the bioavailability and therapeutic efficacy of drugs, potentially transforming the therapeutic landscape for SCI. This review aims to explore the applications and future prospects of nanocarrier-mediated drug delivery systems in the context of spinal cord injury treatment (Figure 2).

## Hydrogel-based nano-biomaterials for SCI treatment

Hydrogels are three-dimensional networks of crosslinked polymers that can hold a large amount of water. Nanoparticles can be incorporated into hydrogels to improve their mechanical properties, drug-delivery capabilities, and cellular interactions. These hydrogels can provide a supportive environment for cell growth, facilitate neural tissue regeneration, and deliver therapeutic agents at the injury site (Zhu et al., 2023; Yin et al., 2024; Liu et al., 2022). When combined with nanomaterials, hydrogels can form a versatile platform for drug delivery and tissue regeneration in SCI treatment (Anderson et al., 2018; Koffler et al., 2019; Liu et al., 2023). The main advantages of using hydrogel-based nano-biomaterials is their ability to provide localized and sustained drug release. By encapsulating therapeutic agents within the hydrogel matrix, drugs can be released in a controlled manner, allowing for long-term treatment and minimizing side effects (Fan et al., 2022; Wang S. et al., 2023).

New types of nano-biological materials have become highly promising tools for the treatment of spinal cord injury (SCI) (Afsartala et al., 2023; Sun et al., 2023). These materials combine the advantages of nanotechnology and biomaterials, which create a supportive environment for tissue nerve regeneration, and functional recovery at the spinal cord injury site. Among these, Among them, hydrogels stand out due to their softness, flexible responsiveness, exceptional absorbency, biocompatibility, chemical stability, and potential applications in tissue engineering (Afsartala et al., 2023; Guan et al., 2024). Hydrogels are classified into two main types: traditional and responsive. Responsive hydrogels react to stimuli such as light, temperature, magnetism, pH, ultrasound, ionic strength, reactive oxygen species (ROS), and glutathione. They can be designed to respond to one or multiple stimuli as a trigger response for controlled drug delivery. They can mimic the three-dimensional structure and scaffolding function of the extracellular matrix. This supports stem cell attachment, proliferation, and differentiation, as well as the release of growth factors, which helps improve the microenvironment after spinal cord injury. Hence, It promots nerve regeneration and acts as a local reservoir for sustained release of drugs or growth factors, continuously enhancing neuronal and tissue regeneration, as well as synaptic regeneration (Han et al., 2022; Roh et al., 2023; Grijalvo et al., 2019). Recently, they have found widespread use in tissue engineering, biomedical, and pharmaceutical field. So providing effective carriers for minimally invasive treatments, precisely filling defects to adapt to the shape and size of injuries, thus promoting tissue repair and inducing regeneration of damaged areas in the body (Yuan et al., 2022; Assuncao-Silva et al., 2015).

Stem cell transplantation using biomaterial scaffolds holds promise for treating SCI. Biomaterial scaffolds can provide a 3D growth environment for mesenchymal stem cells and promote tissue regeneration (Yuan et al., 2022; Qiu et al., 2023). Hyaluronic acid (HA), which is derived from the natural extracellular matrix (ECM), can inhibit glial scar formation, which is beneficial for spinal cord tissue repair. HA hydrogels have a porous structure and a texture similar to spinal cord tissue, giving researchers enough room to design and build three-dimensional cross-linked networks, endowing them with various regulatory functions (Fan et al., 2024; Duarte et al., 2024). Researchers introduced manganese dioxide nanoparticles (MnO_2_NPs) into HA-peptide hydrogels, utilizing the property of MnO_2_, which decomposes hydrogen peroxide (H_2_O_2_) to produce oxygen (O_2_), thereby improving the oxidative stress environment and effectively increasing the survival rate of stem cells. Biomimetic hydrogels based on hyaluronic acid and silk fibroin can keep releasing neurotrophic factors, which really helps to improve recovery after spinal cord injuries (Li et al., 2019). Hydrogels are widely used to construct nerve guidance conduits (NGCs) that direct peripheral nerve repair. Studies have found that conduits combining black phosphorus hydrogels with nerve growth factor (NGF-1) can significantly enhance peripheral nerve regeneration. These conduits strongly induce cell growth, promote Schwann cell proliferation, and facilitate nerve branching. (Hopf et al., 2020; Luo et al., 2021). Hydrogels also play an important role in the repair of spinal cord injuries. By embedding polypyrrole nanoparticles with antioxidant and conductive functions into bioactive hydrogels, the transmission of bioelectrical signals can be restored effectively, promoting nerve regeneration and functional recovery (Luo et al., 2021). Additionally, a temperature-responsive hydrogel system has been developed that can continuously release extracellular vesicles from adipose-derived stem cells *in vivo*. This can greatly boost the movement and growth of Schwann cells, thereby improving the efficiency of nerve regeneration (Zhu et al., 2023; Hopf et al., 2020). The design of hydrogels can also achieve functionalization by adjusting their chemical composition. For example, hydrogels constructed from amino acid derivatives exhibit good biocompatibility and neuroprotective effects, effectively promoting the growth and differentiation of nerve cells (Park et al., 2010; Chen et al., 2024).

In the treatment of spinal cord injuries, dendritic polymers not only serve as drug carriers but also enhance treatment outcome by regulating the drug release rate. For instance, some studies have created hydrogel systems using dendritic polymers that allow for sustained drug release, which boosts the drug concentration at the target site and lowers the chances of systemic side effects (Woerly et al., 2001; Paleos et al., 2007). Additionally, dendritic polymers can work alongside other treatments, such as gene therapy, to improve nerve repair and regeneration after a spinal cord injury.

Furthermore, the application of conductive hydrogels has shown promising prospects in nerve regeneration, as these materials can promote the growth and functional recovery of nerve cells by mimicking the transmission of bioelectrical signals. In terms of wound healing and hemostasis for spinal cord injuries, as well as antibacterial and anti-inflammatory effects to reduce postoperative infection complications, organic gels can be used for long-acting drug formulations to improve drug bioavailability and control drug release, which is very important and has a lot of potential use in long-lasting drug delivery systems.

## Micelles systems for SCI treatment

Micelles are one of the earliest carriers among in drug delivery systems (Dutt et al., 2023; Bose et al., 2021). Currently, the micelles mostly studied are formed from amphiphilic polymer materials and are also known as polymeric micelles (PMs) (Ghezzi et al., 2021). As a type of freely assembled colloid, micelles have a wide range of applications in drug delivery. In recent years, designing polymer materials as carriers for poorly soluble drugs have become hot research topics. With the development of various new amphiphilic block copolymers, micelle-based drug delivery systems have emerged as one of the best options for treating spinal cord injuries (Liu et al., 2017; Feng et al., 2023).

The particle size of micelles generally ranges from 10 to 100 nm. Because of their size and flexibility, they are not easily recognized or captured by the endoplasmic reticulum system (ERS) in the bloodstream. This helps increase the drug’s circulation half-life and prolongs the carrier’s retention time in the blood. They also target the site of spinal cord injury using the Enhanced Permeability and Retention Effect (EPR) effect (Qian et al., 2023; Movassaghian et al., 2015). Polyethylene glycol (PEG), known for its good solubility, is commonly used as an outer protective layer to extend circulation time and create a hydrophilic shell for the micelles (Rasoulianboroujeni et al., 2022; Gill et al., 2015).

Disguising nanomedicine systems as neurotransmitters allows for their delivery to neurons. Researchers coupled the drug CLP-257 with GABA and dopamine (Zuo et al., 2023). CLP-257 is a potassium chloride co-transporter 2 (KCC2) activator that can reduce intracellular chloride ion concentration and decrease neuronal excitability, which reportedly plays a crucial role in motor control. To facilitate minimally invasive delivery and reactive oxygen species (ROS)-responsive release, researchers designed and synthesized amphiphilic block copolymers with PEG hydrophilic segments and hydrophobic segments containing boronic acid ROS scavengers, which encapsulate hydrophobic prodrugs. These micelle-based drugs can be administered intravenously, triggering ROS responses to accumulate and release prodrugs at the injury site (Zuo et al., 2023). This pharmacological approach can target neurons in SCI rat contusion model. It increase axon numbers, further offering neuroprotective effects and enhancing lower limb motor function, bringing new hope for the treatment of SCI and other neurological diseases. Minocycline (MC) has anti-inflammatory, antioxidant, and anti-apoptotic effects in the context of spinal cord injury and also plays a significant role in secondary injury. Polysialic acid (PSA) can promote cell migration and facilitate neuronal and synaptic reconstruction. Polysialic acid-modified (PSM) can significantly protect neurons and myelin from damage, reduce glial scar formation, and recruit endogenous neural stem cells to the lesion site. Thus promoting neuronal regeneration and extending axons, significantly improving rats’ motor function and demonstrating therapeutic efficacy (Wang et al., 2021). Notably, using minocycline alone can lead to hepatotoxic and nephrotoxic effects; however, the PMS drug delivery system avoids these side effects and effectively reduces toxicity. The application of micelle drug delivery systems in spinal cord injury SCI offers a new strategy for drug therapy, with the potential to enhance treatment efficacy and improve patients’ quality of life.

## Nanoparticles systems for SCI treatment

The non-specific distribution and uncontrolled release efficiency of drugs in conventional drug delivery systems (CDDSs) have prompted the design and development of smart drug delivery systems (SDDSs) (Adepu and Ramakrishna, 2021; Hossen et al., 2019). Nanodrug delivery systems can selectively deliver drugs to the site of spinal cord injury, significantly improving drug concentration distribution and release efficiency. Drugs can achieve controlled release in terms of timing and location, and nanodrug delivery systems can enhance drug loading efficiency and bioavailability *in vivo*, improve drug solubility, and reduce side effects. They can also achieve targeted delivery through various administration routes, such as local injection, intravenous injection, and biomaterial-assisted delivery (Rossi et al., 2013; Liu et al., 2020; De Jong and Borm, 2008).

The blood-spinal cord barrier (BSCB) represents a distinct adaptation of the blood-brain barrier (BBB) specifically within the spinal cord area. It is formed by spinal microvascular endothelial cells that are interconnected through tight junctions, along with a basement membrane, pericytes, and the end-feet of astrocytes. The BSCB meticulously controls the ingress and egress of various substances into the spinal parenchyma via a mechanism of selective permeability, thereby preserving the proper functioning of neurons (Rust et al., 2025). Surface modification is also one effective strategy to enhance targeting. Researchers have used human mesenchymal stem cell membrane modification that can effectively penetrate the blood-spinal cord barrier (BSCB) and release drugs at the injury site, promoting axon regeneration and improving the inflammatory environment (Blagovic et al., 2013).

Metal nanoparticles, such as gold nanoparticles, iron oxide nanoparticles, and manganese nanoparticles, have also shown great potential in targeted therapy and imaging. You can achieve high-sensitivity imaging of cells in spinal cord injury models (Chomoucka et al., 2010; Mousavi et al., 2020). Additionally, gold nanoparticles can generate hydrogen gas through catalytic reactions and possess selective antioxidant properties, which help with inflammation during the acute phase of spinal cord injury, thereby improving and enhancing motor function (You et al., 2024). Iron nanoparticles have magnetic properties, and iron oxide nanoparticles containing strontium can improve the imaging quality of magnetic resonance imaging (MRI). The advantage of iron nanoparticles as drug carriers is their ability to enable targeted drug release through an external magnetic field, reducing the impact on healthy tissues. They also have a thermal effect that, when used with photothermal therapy, can boost treatment effectiveness (Dadfar et al., 2019). Manganese nanoparticles can deliver poorly soluble resveratrol to the site of spinal cord injury, and experimental results show that they can inhibit inflammatory factors and apoptotic factors, thereby further promoting motor function in mice. Biocompatible manganese-iron Prussian blue nanoparticles are a new kind of drug carrier and have also demonstrated good immunotherapy effects (Jiang et al., 2022). TiO2/iron nanocomposites are used to load antibiotics and other drugs to make them more bioavailable and targeted. Studies have shown that antibiotics encapsulated in TiO2/iron nanocomposites exhibit excellent antibacterial activity in in vitro experiments, effectively inhibiting the growth of multidrug-resistant bacteria (Zafar et al., 2022; Koli et al., 2016).

## Lipid nanoparticle systems for SCI treatment

Nanoliposomes are nanoscale carriers composed of lipid bilayers, which mainly consist of phospholipids, cholesterol, and other surfactants (Talens-Visconti et al., 2022; Tereshkina et al., 2022). The choice of phospholipids significantly affects the stability, drug encapsulation capacity, and release characteristics of nanoliposomes. The structure of nanoliposomes is typically spherical, with a diameter generally ranging from 50 to 200 nm, exhibiting good biocompatibility and adjustable drug release properties (Mozafari, 2010). Additionally, the surface of nanoliposomes can be chemically modified to enhance their targeting and biocompatibility, for example, by using amino acids or polymer coatings to improve their stability and cellular uptake in biological environments. The breakdown products of nanoliposomes are typically non-toxic and can be safely eliminated from the body, thus providing assurance for their promotion in clinical applications (Hallaj-Nezhadi and Hassan, 2015; Zoghi et al., 2018).

In the treatment of spinal cord injuries, lipid nanoparticle delivery systems are advantageous because they improve drug solubility and bioavailability, overcoming the limitations of traditional drug delivery methods. The design of lipid nanoparticles allows for prolonged drug release *in vivo*, reducing toxic reactions and achieving targeted delivery directly to the injured tissue. These nanoparticles can deliver not only small molecule drugs but also large biomolecules such as proteins and nucleic acids to promote nerve regeneration and repair (Yin et al., 2023). Furthermore, lipid nanoparticle delivery systems also help regulate the microenvironment following spinal cord injury. Research has shown that delivering anti-inflammatory proteins or neurotrophic factors via lipid nanoparticles can significantly reduce the inflammatory response at the injury site, promoting the survival and regeneration of nerve cells (Gao X. et al., 2022). For example, lipid nanoparticles that encapsulate mRNA for human interleukin-10 (hIL-10) can effectively promote neuroprotection and functional recovery. This new delivery system not only improves targeted drug delivery efficiency but also offers new strategies for treating spinal cord injuries. Effectively encapsulating siRNA in nanoliposomes allows for sustained gene silencing at the injury site, thereby regulating the regeneration and repair mechanisms of nerve cells (Gal et al., 2023).

## Synergistic multi-target strategies for SCI treatment

In the treatment of spinal cord injury, traditional therapeutic strategies have often focused on single-target interventions, such as administering anti-inflammatory drugs or neurotrophic factors. While this approach can alleviate post-injury inflammation or promote nerve regeneration to some extent, these isolated interventions frequently fail to fully address the complex pathological processes involved in spinal cord injury. Such injuries not only cause direct mechanical damage but also trigger a cascade of secondary injuries, including oxidative stress, inflammatory responses, and disruption of the blood-spinal cord barrier. These pathophysiological changes mutually interact and exacerbate the extent of neural damage (Xiong et al., 2024; Zuo et al., 2023). Therefore, current therapeutic strategies need to shift toward comprehensive and multi-target interventions, combining different drugs or treatment approaches to effectively intervene at various pathological stages of spinal cord injury (Shi et al., 2025; Qin et al., 2024). Additionally, strategies combining small-molecule drugs and cell therapy have demonstrated potential for improving functional recovery following spinal cord injury. The approach of using small-molecule therapeutics (e.g., antioxidants) in conjunction with stem cell transplantation can effectively mitigate oxidative stress and inflammatory responses while promoting nerve regeneration and functional restoration (Cui et al., 2024; Xu et al., 2023). Research indicates that stem cells possess the potential for self-renewal and differentiation into multiple cell types, enabling them to promote nerve regeneration and functional recovery following spinal cord injury. Human umbilical cord-derived mesenchymal stem cells (hUC-MSCs) can enhance neuronal survival through the secretion of neurotrophic factors and improve motor function after spinal cord injury (Somredngan et al., 2023). In addition, hUC-MSCs can promote the proliferation and differentiation of neural stem cells through specific signaling pathways, such as the Wnt signal pathway, thereby enhancing the effect of nerve regeneration (Wang X. et al., 2023). In addition, using biomaterials as scaffolds not only provides structural support but also continuously releases growth factors, thereby generating positive biological responses in the local microenvironment and promoting cell survival and regeneration (Zhang et al., 2024).

The treatment of spinal cord injury is shifting towards acellular strategies, with engineered extracellular vesicles (EVs) emerging as promising candidates due to their low toxicity, immunogenicity, and ability to transport bioactive molecules across the blood-spinal cord barrier (Qin et al., 2024; Jiang et al., 2020; Kong et al., 2025).

Gene silencing tools play an important role in the treatment of spinal cord injury, especially small interfering RNA (siRNA) and the CRISPR-Cas9 system. They can target and inhibit the expression of specific genes through specific mechanisms, thereby alleviating pathological changes after spinal cord injury. Hydrogel-loaded mesenchymal stem cells (MSCs) and siRNA targeting glial fibrillary acidic protein (GFAP). This combination not only inhibits scar formation but also promotes neurogenesis, thereby creating a more favorable microenvironment for the regeneration of nerve cells and enhancing the therapeutic effect on the injured area (Zhang et al., 2025; Kim et al., 2021).

## Clinical translational bottlenecks and strategies

In the clinical translation of nanoparticle delivery systems, several key factors affect their efficacy. Firstly, the biocompatibility and safety of nanoparticles are fundamental requirements for clinical applications. Research shows that the composition, surface properties, and release characteristics of nanoparticles can influence their behavior *in vivo* (Matson et al., 2022). Therefore, selecting appropriate biomaterials as nanoparticle carriers and functionalizing their surfaces to enhance their stability and targeting *in vivo* is one of the optimization strategies (Zhu et al., 2025).

In the treatment of spinal cord injury, multi-modal therapeutic strategies are gradually emerging. Methods such as stem cell therapy, biomaterial scaffolds, nano-delivery systems, and targeted molecular therapy show good potential for clinical application. These strategies not only promote the regeneration and repair of neurons but also improve the microenvironment, making the recovery process at the injury site smoother. The results of clinical trial NCT01321333 indicate that the safety of using stem cell therapy for SCI has been preliminarily validated, but the efficacy still needs further confirmation (Guo et al., 2021). However, there may be controversies regarding the results and opinions of different studies on therapeutic effects, as some therapies have not been as effective as expected in certain clinical trials.

Additionally, the drug loading efficiency and release mechanism are also important factors affecting therapeutic effects. By optimizing the preparation process of nanoparticles, large-scale production, and quality control, the drug encapsulation rate can be improved, ensuring that sufficient drug concentration reaches the target. Furthermore, adjusting the release rate of nanoparticles to match the pathological process is also an effective optimization strategy (Wang and Bai, 2024).

Moreover, the design and implementation of clinical trials are also key to achieving the clinical transformation of nanotechnology. It is necessary to design reasonable clinical trials to evaluate the safety and efficacy of nanodelivery systems, while considering individual differences among patients. At the same time, integrating the advantages of biomedical engineering, medicinal chemistry, and clinical medicine through interdisciplinary collaboration will help promote the application of nanotechnology in the treatment of spinal cord injuries (Matson et al., 2022; Zhu et al., 2025).

Finally, market access and regulatory approval are also important factors affecting the clinical application of nanodelivery systems. With the rapid development of nanotechnology, relevant regulatory policies and standards need to be continuously updated to ensure the safety and efficacy of nanomedicines, paving the way for clinical applications (Wang and Bai, 2024). In the future, the field of spinal cord injury treatment needs to strengthen the integration of mechanism analysis and clinical translation. By deeply exploring the molecular mechanisms after injury and the mechanisms of different treatment strategies, clearer guidance can be provided for clinical practice. At the same time, optimizing treatment plans to maximize the recovery of function in spinal cord injury patients is a common goal for researchers. Only through interdisciplinary collaboration and continuous exploration can more effective treatment options and better prognoses be provided for spinal cord injury patients.

## Conclusion

SCI is a series of complex and dynamic changes, with high diversity for different patients. Changes in the post-injury microenvironment has yielded information showing the value and essential role played by neuroprotective and neurodegenerative therapies for clinical and basic research. In the future, we should comprehensively utilize multi-target positioning strategies that address both inherently edematous conditions and the complex disease processes associated with SCI. This includes targeting external cellular pathways and mediators within the SCI microenvironment to develop personalized treatment programs.

In recent years, nanomaterials have shown exceptional potential and high probability for applications in the field of SCI. These materials, through their unique physicochemical properties, can effectively promote cell regeneration, improve the inflammatory microenvironment, and enhance motor function, offering new hope for treatment of patients with SCI. Various methods, including cell-targeted drug delivery, tissue-specific targeting, and receptor-mediated endocytosis, can effectively increase drug selectivity. These methods also help reduce the impact on healthy cells. Cell-targeted drug delivery refers to the direct delivery of drugs or therapeutic substances into specific types of cells via specific carriers or molecules. For example, using stem cells or immune cells as drug carriers can achieve precise delivery by specifically binding to target cells. Tissue-specific targeting refers to optimizing the properties of drug carriers so that they can selectively accumulate in specific tissues or organs. Receptor-mediated endocytosis is the process by which cells recognize and uptake exogenous molecules through specific receptors. This mechanism is very important in drug delivery, especially in targeted therapy. By designing drug carriers that can specifically bind to receptors on the surface of target cells, effective endocytosis and cell uptake can be achieved. For instance, using antibody-drug conjugates (ADCs) allows for direct delivery of drugs into cancer cells, thereby enhancing therapeutic effects and reducing damage to normal cells. With the rapid development of research technologies and the deep integration of medical and engineering disciplines, various new nanodrug delivery systems have emerged. These will surely play an important role in the future of the spinal cord injuries.
